# Gaucher disease in a patient with membranoproliferative glomerulonephritis: case report

**DOI:** 10.1186/s12882-023-03163-9

**Published:** 2023-09-29

**Authors:** Mengjun Liang, Shiyan Zhu, Shaoqin Liu, Jianquan Chen, Danni Li, Chengzhi Luo, Xiaowen Wang, Zongpei Jiang

**Affiliations:** 1 Department of Nephrology, The Sixth Affiliated Hospital, Sun Yat-sen University, 26 Yuancun Er Heng Road, 510655 Guangzhou, China; 2 Department of Nephrology, The Third Affiliated Hospital of Guangdong Medical University, 39th, Donghua Road, Longjiang, Foshan China

**Keywords:** Gaucher disease, Membranoproliferative glomerulonephritis, Prednisone, Mycophenolate mofetil, Case report

## Abstract

**Background:**

Gaucher disease (GD) is a rare autosomal recessive inherited, lysosomal storage disoder that involves liver, spleen, lung, bone, bone marrow even central nervous. However, GD associated membranoproliferative glomerulonephritis (MPGN) is seldom reported.

**Case presentation:**

Here we described a case of 35-year-old man suffering from GD with hepatosplenomegaly, ascites, bone destruction, myelofibrosis and MPGN. Renal biopsy revealed MPGN and Gaucher cells presented in the glomeruli capillaries. β-glucosidase activity was 1.95nmol/1 h/mg and gene detection demonstrated that one homozygous pathogenic variant Leu483Pro in GBA. He received the treatment of oral prednisone and mycophenolate mofetil and his ascites and renal outcomes had been significantly improved.

**Conclusions:**

Therapy of prednisone and mycophenolate mofetil may be an optional choice for patients with Gaucher disease who have no opportunity to use enzyme treatment.

## Background

Gaucher disease (GD) is a rare, autosomal recessive genetic disorder. The incidence of GD ranged from 0.70 to 1.75 per 100,000, but it could reach 118 per 100,000 in the Ashkenazi Jewish population [1, 2]. It is caused by deficiency of the lysosomal enzyme, glucocerebrosidase (β-glucosidase) and accumulation of glucosylceramide in macrophages (Gaucher cells). The lysosomal storage disease would involve liver, spleen, lung, bone, bone marrow even central nervous [3]. Enzyme replacement therapy (ERT) and substrate reduction therapy (SRT) have been universally accepted as the specific therapy for GD [3].

Renal involvement in GD patients is rarely reported in contrary to other storage diseases such as Fabry disease [4]. Here we described a case of 35-year-old man suffering from GD with hepatosplenomegaly, ascites, myelofibrosis and MPGN and he received an effective treatment of oral prednisone and mycophenolate mofetil.

This study was conducted in accordance with the Declaration of Helsinki and approved by the institutional review board of The Third Affiliated Hospital of Guangdong Medical University (2020(024)). Written informed consent was obtained from this patient.

## Case presentation

A 35-year-old man with refractory abdominal bloating for more than 20 years, splenectomy at 21 years old for splenomegaly and weekly paracenteses for ascites during the past 5 years which had been diagnosed liver cirrhosis, was characterized by 1-month history of edema and heavy proteinuria, without fever, dyspnea or oliguria. On admission, the temperature was 36.5℃, blood pressure was 120/70mmHg, and there was an enlarged liver and positive shifting dullness. He had the height of 155 cm with thoracolumbar scoliosis deformity and moderate edema of bilateral lower limbs.

**Fig. 1:**
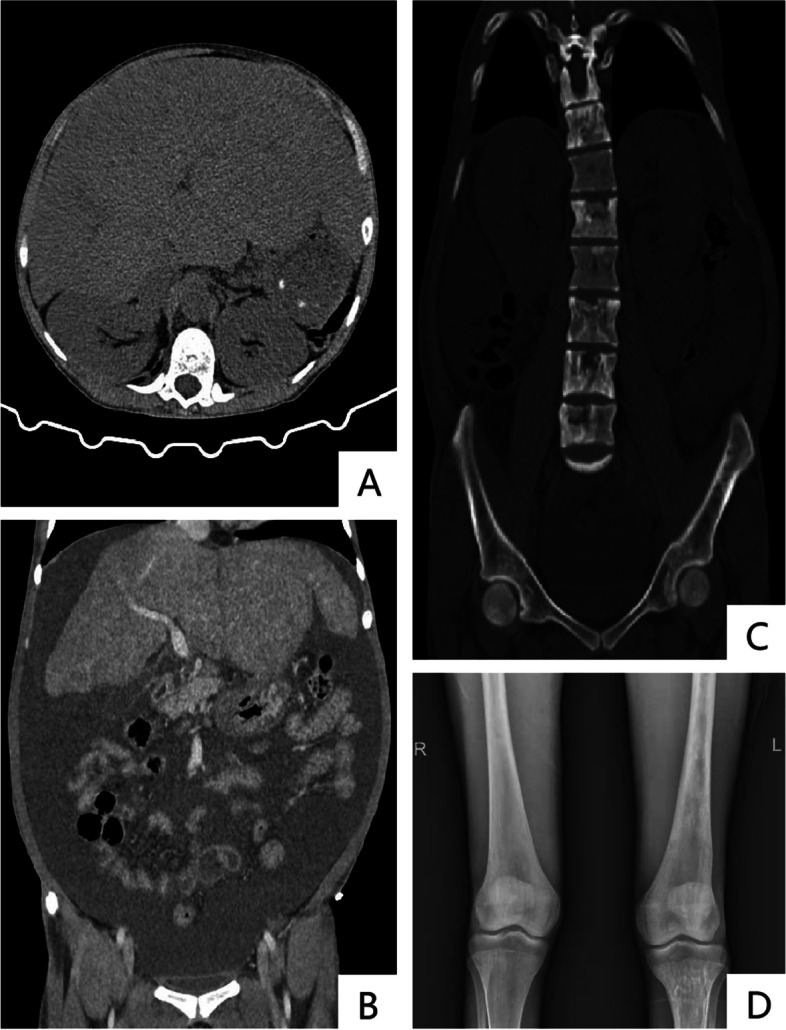
**A** CT scan showed diffusely enlarged liver. **B** CT scan showed abundant ascites. **C** CT scan showed bone destruction in multiple thoracolumbosacral vertebrae especially in sacrum and left ilium. **D** Femoral radiographs showed decreased bone density and symmetrical widening of middle and lower segments of both femurs, which looked like the "beer bottle shape" changed. CT=Computed tomography

 Initial laboratory values listed in Table 1. Ascites routine revealed total protein of 7.2 g/L and WBC count of 85*10^6^/L. No remarkable findings were found in important secondary causes, including diabetes mellitus, systemic lupus erythematosus, autoimmune hepatitis, hepatitis B and C, human immunodeficiency virus, carcinoma, multiple myeloma, antineutrophil cytoplasmic antibody (ANCA)-associated vasculitis, anti-glomerular basement membrane (anti-GBM) antibody or anti-PLA2R antibody assay. Thoracoabdominal computed tomography (CT) scan showed: diffusely enlarged liver, absent spleen, abundant ascites and bone destruction in multiple thoracolumbosacral vertebrae especially in sacrum and left ilium. Radiographs showed decreased bone density and symmetrical widening of lower segments of both femurs, which looked like the “beer bottle shape” changed (Fig. 1).

**Table 1:** Initial laboratory values on admission

Lab. tests	Values
White blood cell (WBC)	17.7*10^9^/L
Red blood cell (RBC)	4.0*10^12^/L
Hemoglobin	118 g/L
Thrombocyte (PLT)	271*10^9^/L
Serum creatinine (SCr)	144µmol/L
eGFR (CKD-EPI formula)	54ml/min/1.83m^2^
Serum albumin (Alb)	12 g/L
Serum globulin (Glb)	42.9 g/L
Serum IgG	19.0 g/L
Serum complement C3	0.8 g/L
Serum complement C4	0.2 g/L
24-hour urine protein	5.8 g/1500ml
Microscopic hematuria	+++

**Fig. 2:**
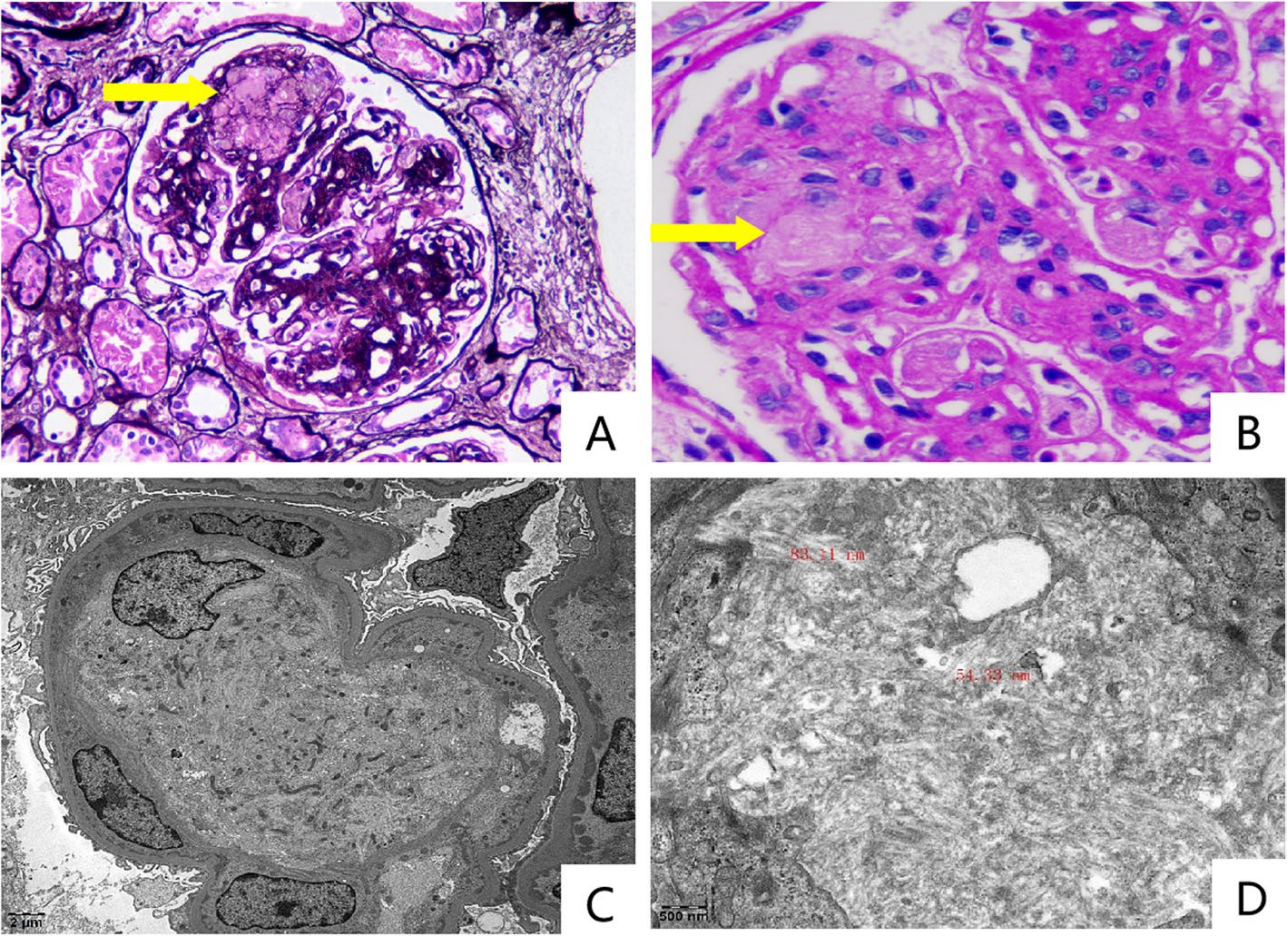
**A** Glomerular mesangial cells and matrix showed severe diffuse hyperplasia; basement membrane was thickened; mesangial matrix was inserted and two-track sign was formed. Gaucher cells were seen in the glomerular capillary. (yellow arrow) (Periodic acid-methenamine silver original magnification×200) (**B**) Gaucher cells with “crumpled tissue paper” appearance in the glomerular capillary. (yellow arrow) (Periodic acid-schiff stain original magnification×400) (**C**) EM showed segmental thickening of the basement membrane (about 400-900nm), with segmental mesangial insertion. Electron-dense deposits consisting of both immunoglobulin and C3 were detected in the subendothelial and mesangial regions. Monocytes/macrophages were seen in the lumen of the capillary in the glomerulus. **D** Twisted microtubules with diameters of approximately 50-80nm could be seen in the lumen of glomerular capillary (red labels). EM=Electron microscope

 The renal biopsy revealed membranoproliferative glomerulonephritis (MPGN) with infiltrating intracapillary macrophages that exhibit a “crumpled tissue paper” appearance typical of Gaucher cells. Light microscopy also showed 65% tubulointerstitial scarring with multifocal lymphohistiocytic infiltrates. Immunofluorescence showed subendothelial and mesangial staining for IgG (+++), IgA (+), IgM (++), C3 (+++), Clq (+++), Kappa (±), and Lambda (+). IgG subtype panel showed staining for IgG1(+), IgG2(+), IgG3(+++), and IgG4(+). Electron microscope (EM) showed segmental thickening of the basement membrane (about 400-900 nm). Twisted microtubules with diameters of approximately 50-80 nm could be found inglomerular capillary (Fig. 2). A bone marrow biopsy revealed myelofibrosis. No gaucher cells were identified and JAK2-V617F was negative.

Further assessment of β-glucosidase activity was 1.95nmol/1 h/mg (> 6.8nmol/1 h/mg for normal reference, Colorimetric method) and gene detection demonstrated that one homozygous pathogenic variant c.1448T > C in 1q22, Exon11, p.(Leu483Pro) which was a missense mutation. .

**Fig. 3:**
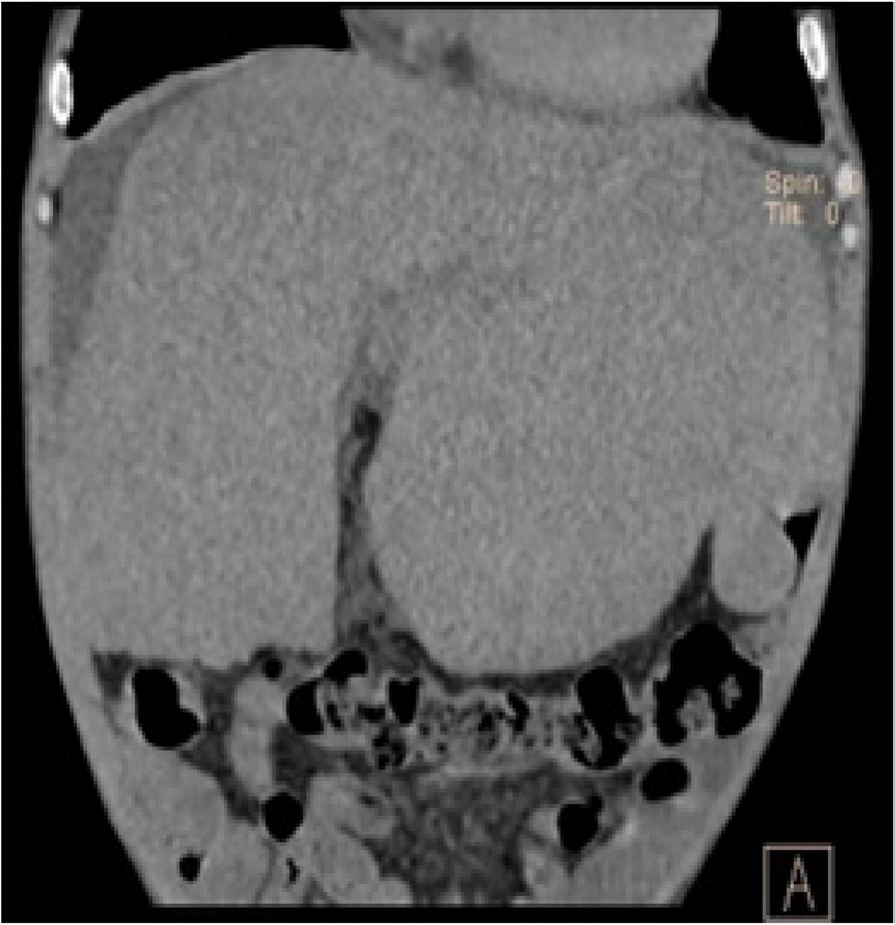
Abdominal CT showed persistent enlarged liver but few ascites after 8-week follow-up. CT=Computed tomography

 He received the treatment of oral prednisone (0.5 mg/Kg/d) and mycophenolate mofetil (MMF, 0.5 g bid). The abdominal CT scan showed persistent enlarged liver but few ascites after 8 weeks therapy (Fig. 3). Upon 12-week follow-up, his Scr decreased to 82 µmol/L (eGFR 106ml/min/1.83m^2^); serum Alb increased to 27 g/L; urine protein decreased to 0.8 g/24 h and the activity of β-glucosidase was 1.34nmol/1 h/mg. There was no adverse effects happened on the patient.

## Discussion and conclusions

Gaucher disease (GD), first described by Philippe Gaucher in 1882, is an autosomal-recessive disorder due to a confirmed lysosomal enzyme (β-glucosidase) deficiency caused by mutations in the GBA gene, located on chromosome 1 and it contains 11 exons [3]. More than 400 mutations have been described in the GBA gene, but some of them are more common, such as L444P (c.1448T > C) named Leu483Pro in Human Genome Variation Society (HGVS) - recommended nomenclature [5, 6]. There are three clinical forms identified. Type 1 (non-neuronopathic form) is the classic phenotype and can be diagnosed at any age, with the incidence of 85−95% [6, 7]. Types 2 and 3 (neuronopathic Gaucher Disease, NGD) are characterized by neurological impairment and both rare and severe with different progression; type 2 is termed acute NGD with the incidence of 4−20% and types 3 is named subacute or chronic NGD with the incidence of 5−30% [7, 8].

In our case, the 35-year-old man had suffered from GD, onset before 15 years old, with hepatosplenomegaly, splenectomy, ascites, bone destruction, myelofibrosis and MPGN. The activity of β-glucosidase was decreased, 1.95nmol/1 h/mg and gene detection demonstrated variant Leu483Pro. The diagnosis of type 1 GD (GD1) was verified.

Splenomegaly is observed in more than 90% of GD patients and hepatomegaly is noted in 60-80% of GD patients [3]. Persistent liver enlargement, with or without liver enzymes alteration, fibrosis, cirrhosis and portal hypertension, is the consequence of intra-hepatic accumulation of Gaucher cells and secondary inflammatory response. Portal hypertension in GD is not just secondary to the presence of liver cirrhosis, since the overflow in the portal system secondary to splenomegaly or the massive infiltration of Gaucher cells in liver parenchyma especially in splenectomized patients [9, 10]. In this case, peritoneal tap for ascites and its routine test indicated that it was non-inflammatory, which was thought to be related to portal hypertension and hypoalbuminemia.

Bone involvement is another common disorder in GD, with the prevalance of 76-94% of in GD1 patients, with the skeletal manifestations including bone mass destruction and bone marrow infiltration. Radiological findings include Erlenmeyer flask deformity, osteopenia, osteosclerosis, osteonecrosis, and fractures, predominantly in the pelvis and lower limbs (more rarely in the upper limbs) [11, 12]. As the previous report, GD and primary myelofibrosis has shared similar clinical and laboratory features, such as hepatosplenomegaly and marrow fibrosis, often resulting in a misdiagnosis [13]. Some variables like onset age, clinical manifestation and negative JAK2-V617F, a mandatory diagnostic tool in myeloproliferative neoplasm [14], played a role in the differential diagnosis.

Although other storage diseases, such as Fabry disease, frequently affect the kidneys, a systematic evaluation has not found renal abnormalities in patients with GD [15]. Up to now, only a few cases on GD related glomerulopathies have been reported, such as focal and segmental glomerulosclerosis (FSGS) [16, 17], mesangiocapillary glomerulonephritis because of aseptic necrosis of the femoral heads [18], preeclampsia-induced glomerular endotheliosis [19], renal amyloidosis [20] and renal failure due to multiple myeloma [21]. According to the cases above, a common pathological finding is the accumulation of Gaucher cells, which are macrophages and monocytes essentially, in glomeruli or interstitium. Kidney abnormality in GD is speculated to involve immune system disorder, which may be related to reduced inactivation of Igs due to liver disorder, or combined with hematological malignancy.

The case we presented here is diagnosed as GD related MPGN, which has never been reported before. Typical pathological characteristics in renal biopsy showed “crumpled tissue paper” appearance typical of Gaucher cells were thought to be the compelling evidence for the diagnosis of GD related MPGN. Moreover, the findings showed an immune complex mediated MPGN.

Treatment of MPGN or ICGN depends on the identification of the underlying cause [22]. ERT and SRT have been universally accepted as the specific therapy for GD [3]. As the previous literature, common therapeutic strategies for renal involvement in GD included ERT, supportive treatment or renal replacement therapy for patients with renal failure, but no evidence of effectiveness on glucocorticoid [16–21]. This patient refused ERT because of his poor economic situation.

According to the 2021 Kidney Disease: Improving Global Outcomes (KDIGO), for patients with idiopathic ICGN, abnormal kidney function (without crescentic formation), active urine sediment, with or without nephrotic-range proteinuria, glucocorticoids and immunosuppressive therapy, as oral MMF or cyclophosphamide plus low-dose glucocorticoids, has been suggested [22]. Actually, in the present case, the patient had received the treatment of oral prednisone and MMF and his renal outcomes and ascites were significantly improved. Remission of proteinuria and improvement of hypoalbuminemia would do good to reduction of ascites. On the other hand, whether prednisone and MMF would play a role in lowering portal pressure which was thought to be another important cause of ascites? Recent studies have confirmed glucocorticoid as the recommended therapy in severe alcoholic hepatitis and mycophenolate mofetil as first-line treatment of autoimmune hepatitis for their inhibition effect on liver immune response [23, 24]. Unfortunately, we had not completed portal pressure measurement before treatment and we would follow up the case for more evidence.

Here we presented a case of 35-year-old man suffering from GD1 with hepatosplenomegaly, ascites, bone destruction, myelofibrosis and MPGN. He received the treatment of oral glucocorticoid and MMF, which improved the liver and renal outcomes. The case provided us more therapeutic strategies for the GD1 patients lost to ERT therapy according to their characteristic clinical manifestations. Therapy of prednisone and mycophenolate mofetil may be an optional choice for patients with Gaucher disease who have no opportunity to use enzyme treatment.

## Data Availability

All data generated or analysed during this study are included in this published article.

## Abbreviations

GD: Gaucher disease
MPGN: Membranoproliferative glomerulonephritis
ERT: Enzyme replacement therapy
SRT: Substrate reduction therapy
WBC: White blood cell
RBC: Red blood cell
SCr: Serum creatinine
Alb: Serum albumin
Glb: Serum globulin
ANCA: Antineutrophil cytoplasmic antibody
anti-GBM: Anti-glomerular basement membrane
CT: Thoracoabdominal computed tomography
Igs: Immunoglobulin
MMF: Mycophenolate mofetil
HGVS: Human Genome Variation Society
NGD: Neuronopathic Gaucher Disease
ICGN: Immune complex-mediated glomerulonephritis
KDIGO: Kidney Disease Improving Global Outcomes
